# Immune Response to Coccidioidomycosis and the Development of a Vaccine

**DOI:** 10.3390/microorganisms5010013

**Published:** 2017-03-16

**Authors:** Natalia Castro-Lopez, Chiung-Yu Hung

**Affiliations:** 1 Department of Biology and South Texas Center for Emerging Infectious Diseases, University of Texas at San Antonio, San Antonio, TX 78249, USA; jpm915@my.utsa.edu; 2 Immune Defense Core, University of Texas, San Antonio, TX 78249, USA

**Keywords:** *Coccidioides*, San Joaquin Valley fever, coccidioidomycosis, fungal infection, innate immunity, *Coccidioides* vaccine, T-cell response

## Abstract

Coccidioidomycosis is a fungal infection caused by *Coccidioides posadasii* and *Coccidioides immitis*. It is estimated that 150,000 new infections occur in the United States each year. The incidence of this infection continues to rise in endemic regions. There is an urgent need for the development of better therapeutic drugs and a vaccine against coccidioidomycosis. This review discusses the features of host innate and adaptive immune responses to *Coccidioides* infection. The focus is on the recent advances in the immune response and host-pathogen interactions, including the recognition of spherules by the host and defining the signal pathways that guide the development of the adaptive T-cell response to *Coccidioides* infection. Also discussed is an update on progress in developing a vaccine against these fungal pathogens.

## 1. Coccidioidomycosis and *Coccidioides*

Coccidioidomycosis, commonly known as San Joaquin Valley fever or Valley fever, is a fungal infection with high morbidity and potential mortality affecting persons in the endemic areas. *Coccidioides* species are endemic to certain arid to semiarid regions of the southwestern United States, northern Mexico, and scattered areas of Central and South America [1]. Recent epidemiological and population studies suggest that the geographic range of coccidioidomycosis is expanding, as new cases have been identified in the state of Washington, well outside the established endemic range [2]. The two etiologic agents are dimorphic *Coccidioides immitis* and *Coccidioides posadasii* which grow in the appropriate soil as fungal mycelia. The fungal mycelia segment into arthroconidia (spores) which are aerosolized and capable of causing infection. Infection usually occurs by inhalation of the airborne spores after disruption of the fungal mycelia in the soil. The inhaled spores first convert to spherule initials which grow in the lungs into multinucleate spherules (20–100 μm diameter) [1,3,4]. Spherules become compartmentalized by ingrowth of networked segmentation septa. Each compartment is further partitioned into a dense cluster of spherical endospores [3]. The spherules mature and eventually burst, releasing hundreds of endospores (2–10 μm diameter). The endospores are then capable of differentiating into new spherules and the parasitic life cycle is repeated. This sequence of morphological events can be reproduced in vitro by growth of the organism in a chemically defined medium, purged with 20% CO_2_/80% air [5]. Both *C. immitis* and *C. posadasii* are highly infectious; probably all mammals that reside in the areas of endemicity are at risk to develop a *Coccidioides* infection. The minimum number of spores needed to cause symptomatic disease in human is not known. However, intranasal inoculation with approximately 10 viable spores to BALB/c mice is sufficient to cause disseminated disease and death in two to three weeks post-challenge [6]. Typically, *Coccidioides* spores transform into spherules in the terminal bronchi and begin to endosporulate in the lungs within two to four days [7]. Endospore release is essential for lymphogenous or hematogenous dissemination of the pathogen within tissues of the host.

## 2. Public Health Impact of Coccidioidomycosis

Coccidioidomycosis consists of a broad spectrum of illness. At one end of the spectrum, it may produce a mild flu-like syndrome or an uncomplicated pneumonia at one to four weeks after inhalation of the fungal spores, either of which may resolve spontaneously. Most infected individuals recover during the subsequent weeks, though recovery may take several months on occasion. At the other end of the spectrum, *Coccidioides* infection can lead to progressive pulmonary destruction or life-threatening, disseminated disease, which may involve skin, bone, muscle, and/or the central nervous system [8]. African-Americans, Hispanics and Pacific Islanders are more susceptible to severe coccidioidomycosis compared to Caucasians. Elderly persons and pregnant women are also at risk for severe disseminated coccidioidomycosis [9]. In recent years, it has become evident that persons with immunodeficiency diseases, diabetes, and those who are transplant recipients are also particularly vulnerable [10,11]. The incidence of reported coccidioidomycosis increased substantially, from 5.3 per 100,000 people in the southwestern US in 1998, to 42.6 per 100,000 in 2011 [12]. Annually, approximately 30,000 new cases of coccidioidomycosis are reported in Arizona and California [12]. It is estimated that the incidence of coccidioidomycosis is 7.6 per 100,000 people in Mexico, and 7.12 cases per 1000 hospitalized admissions in Brazil [13,14]. In endemic areas, 17%–29% of patients who contract pneumonia outside of hospitals or extended care are due to *Coccidioides* infection [15]. During 2000–2011, there were 25,217 coccidioidomycosis-associated hospitalizations and greater than $2 billion USD in total hospital charges in California [16]. Collectively, these statistics highlight the increasing health- and cost-related impacts of coccidioidomycosis-associated hospitalizations as a major public health challenge [17].

## 3. Responses of Innate Immune Cells to *Coccidioides* Infection

The surveillance and elimination of fungal pathogens depend on the sentinel nature of phagocytic cells of the innate immune system, especially macrophages and neutrophils. Phagocytic cells are capable of engulfing arthroconidia, spherule initials, and endospores, but they fail to ingest mature spherules due to their large cell sizes (20–100 μm) [18,19,20]. *Coccidioides* mainly establishes an extracellular relationship with host cells, but an intracellular interaction also exits. Phagocytes expand their surface areas to accommodate large spherules during the frustrated phagocytosis process, causing high stress for cells [20]. It appears that spherule initials and endospores are the most vulnerable to growth inhibition and killing by activated phagocytes. Neutrophils, although short-lived, are the most abundant and rapidly responding phagocytes in the innate immune system. Endospores released from mature spherules trigger an influx of neutrophils to the *Coccidioides* infection sites [18,21]. The chemotactic response of human neutrophils to spherules has been shown to be as robust as *Candida albicans* stimulations [20]. Neutrophils can inhibit the growth of spherule initials and endospores (<10 μm) in vitro [18,22]. Depletion of neutrophils from mice results in accelerated *C. albicans* propagation in tissue and increased mortality [19]. Contrary to this, the role of neutrophils in combating *Coccidioides* infection in vivo depends on whether the host has experienced prior exposure to *Coccidioides*. Neutrophil-depleted, naïve C57BL/6 mice are highly susceptible to primary *Coccidioides* infection in a manner similar to wild-type mice, suggesting neutrophils are not essential for protection against this mycosis [23]. On the other hand, the protective efficacy of a live, attenuated (ΔT) vaccine against respiratory coccidioidomycosis is dependent on neutrophils [23]. Neutrophils may act as double-edged swords, by playing protecting roles in eliminating microbial infections, but the excessive release of oxidants and proteases may be responsible for injury to organs and can cause fungal sepsis [24].

Macrophages also infiltrate microbial infection sites in response to various inflammatory signals. Once within tissues, macrophages further develop into subsets with distinct phenotypes and functions that are designated as classically and alternatively activated macrophages (also known as M1 and M2), respectively [25]. Proinflammatory cytokines (e.g., interferon-γ (IFN-γ)) guide the polarization of M1 macrophages, whereas interleukin (IL)-4 mediates the development of M2 phenotypes. Murine macrophages respond to spherule stimulation by producing proinflammatory chemokines and cytokines, including macrophage inflammatory protein-2 (MIP-2; also known as chemokine (C-X-C motif) ligand 2 (CXCL2)), granulocyte-macrophage colony-stimulating factor (GM-CSF), tumor necrosis factor (TNF)-α, IFN-γ, IL-1β, IL-6, IL-12, IL-17A, IL-22, and IL-23 [26,27]. The ability of macrophages to kill spherule initials and endospores in vitro seems to be dependent on their activation conditions [28]. In the presence of IFN-γ and TNF-α, macrophages are capable of killing *Coccidioides* endospores [29]. In contrast, without these inflammatory cytokines, *Coccidioides* endospores can inhibit the formation of phagolysosomes [28,29]. Furthermore, elevated numbers of macrophages are recruited to the lungs of vaccinated mice compared to non-vaccinated animals after intranasal challenge with *Coccidioides* spores [30]. Macrophages presumably play a role in the clearance of this pathogen, while their phenotypes remain to be characterized. Phagocytic cells are armed with enzymes to produce a chemical arsenal, including reactive oxygen and nitrogen species (ROS and RNS), during respiratory burst. Hydrogen peroxide and superoxide radicals generated by nicotinamide adenine dinucleotide phosphate oxidase (NOX2) and nitric oxide (NO), produced by inducible nitric oxide synthase 2 (iNOS), have been implicated in *Coccidioides* killing. Interestingly, *NOS2*^−/−^, *NOX2*^−/−^ and wild-type mice show comparable percent survival and lung fungal burdens after intranasal challenge with *Coccidioides* spores, indicating that iNOS and NOX2 alone do not play a significant role in the control of *Coccidioides* infection in mice, and the underlying mechanism(s) of the macrophage-mediated response against spherules requires further study [31,32,33].

To survive the attack of phagocytic cells, *Coccidioides* spherules have developed countermeasures aimed at evading phagocytosis or suppressing the production of killing compounds. *Coccidioides* expresses a parasitic phase-specific spherule outer wall glycoprotein (SOWgp) which has been determined to be a major antigen on cell surfaces [34,35]. Opsonization of parasitic cells with a polyclonal anti-SOWgp antibody increases phagocytosis and killing of spherule initials by murine macrophages in vitro [36]. Interestingly, *Coccidioides* endospores can evade phagocytosis by expression of a metalloproteinase (Mep1) to digest SOWgp from their cell surface during the phase of development when these fungal cells are most vulnerable to phagocytic cell defenses [36]. Another tactic is to suppress the production of hydrogen peroxide, hypochlorous acid, and nitric oxide by macrophages [18,22,37]. The low production of these antimicrobial oxidants may contribute to the resistance of *Coccidioides* to phagocytes [22].

Dendritic cells (DCs) are the bridge between the innate and adaptive immune systems. DCs capture and process antigens derived from fungal pathogens, and, upon maturation, they guide the development and differentiation of lymphocytes to initiate the adaptive immune response [38]. DCs play a pivotal role in antigen presentation and activation of lymphocytes during coccidioidomycosis [39]. Toluene spherule lysates of *Coccidioides* can induce maturation of human DCs [39,40]. DCs stimulated with T27K antigen, a soluble protein extract of spherules, are capable of inducing proliferation of peripheral blood monocytic cells (PBMCs) derived from both coccidioidomycosis patients and non-immune donors [41]. Furthermore, cytokine analysis reveals that the stimulated PBMCs produce an elevated level of IFN-γ, which has been associated with protective immunity against coccidioidomycosis [41]. Owing to their immune-stimulating properties, DCs have been developed into an experimental vaccine to protect mice against pulmonary coccidioidomycosis [42,43]. A DC-based vaccine may not be applicable for prophylactic usage due to technical difficulties and high production costs, but it offers a potential therapeutic strategy for patients with severe disseminated coccidioidomycosis.

## 4. Recognition of *Coccidioides* by Pattern Recognition Receptors (PRRs)

The pattern recognition receptors are sensor proteins expressed by cells of the innate immune system. Pattern Recognition Receptors detect evolutionarily conserved structures on microbes, termed pathogen-associated molecular patterns (PAMPs). Four types of PRRs, Toll-like receptors (TLRs), C-type lectin receptors (CLRs), Nod-Like receptors (NODs), and Rig-I like receptors, have been implicated in the recognition of fungal PAMPs [44]. The importance of PRRs in the control of *Coccidioides* infection has been demonstrated in several studies, showing that TLRs and CLRs orchestrate the recognition of spherule wall components to initiate innate immunity and, subsequently, activate adaptive responses against this fungus [23,26,27,45,46,47,48]. Ten human and 12 murine TLRs that recognize and transduce signals via myeloid differentiation primary response protein 88 (MyD88)-dependent and/or MyD88-independent pathways have been characterized thus far [49]. Bone marrow–derived macrophages of mice respond to live and formalin killed spherules (FKS) by producing inflammatory mediators (i.e., TNF-α, MIP-2, and IL-6) in a TLR2-dependent manner [26]. *TLR2*^−/−^ mice are more susceptible to primary *Coccidioides* infection compared to their wild-type counterparts, indicating that TLR2 contributes to the initial recognition of spherules. Both *TLR2*^−/−^ and wild-type WT mice are similarly protected by a live-attenuated (ΔT) vaccine, suggesting that unidentified complementary PRRs are involved in guiding the development of adaptive immunity against coccidioidomycosis [23]. TLR4 may not be required for inducing adaptive immunity, but may be essential for preventing the extrapulmonary dissemination of *Coccidioides* [48].

C-type lectin receptors are a large superfamily of transmembrane proteins, mainly expressed on myeloid cells, including macrophages, dendritic cells, and granulocytes [44]. CLRs recognize microbial carbohydrates, lipids, and proteins via one or more extracellular C-type lectin-like domains (CTLDs). Dectin-1 and Dectin-2, each having an extracellular CTLD, are of particular interest due to their ability to interact with fungal cell wall components. Dectin-1 recognizes fungal β-1,3-glucan exposed on the cell wall and recruits spleen tyrosine kinase directly through its immunoreceptor tyrosine-based activation motif (ITAM). Dectin-2 and macrophage-inducible C-type lectin (Mincle) recognize mannose-like structures. We, and others have shown that soluble fusion proteins, consisting of the extracellular domain of Dectin-1, Dectin-2, and Mincle can bind to spherules [27,45]. We have further constructed reporter cells that express a membrane-bound form of these three CLRs to assess the CLR-mediated response to *Coccidioides* infection [45]. Results show that membrane-bound forms of Dectin-1 and Dectin-2 can also interact with *Coccidioides* and activate reporter gene expression. In contrast, Mincle-expressing reporter cells are not activated by spherule stimulation [45]. In vivo studies using *Dectin-1^−/−^* mice infected with *Coccidioides* have revealed lower levels of Th17 cytokines and modestly increased fungal burden in their lungs compared to wild-type counterparts [27]. Furthermore, macrophages derived from *Dectin-2^−/−^* mice produce reduced amounts of proinflammatory cytokines compared to wild-type mice, though both strains of mice are similarly susceptible to pulmonary coccidioidomycosis [47]. Taken together, these data suggest that Dectin-1 and Dectin-2 cooperate in the innate recognition of *Coccidioides* infection, but that Mincle is not required for activation of the early innate response [45].

## 5. Adaptive T-Cell Responses to *Coccidioides* Infection

Results from animal studies have shown that T-cell immunity is essential for adaptive immunity against coccidioidomycosis [9,50]. There is considerable plasticity in adaptive immunity against fungal pathogens. In the absence of CD4^+^ T helper cells (Th), CD8^+^ T cytosolic cells (Tc) can confer protection in a major histocompatibility complex (MHC) class I–dependent manner [50,51,52,53]. In this review, we focus on the recent progress in the characterization of CD4^+^ T-cell responses to *Coccidioides* infection. CD4^+^ T-cells differentiate into distinct subsets that produce restricted types of cytokines to defend against various microbial pathogens [54]. Known CD4^+^ T-cell subsets include Th1, Th2, Th17, Th9, Th25, T follicular helper cells (Tfh), and regulatory T-cells (Treg) [55]. Many studies of coccidioidomycosis, conducted prior to Th17 discovery, have reported IFN-γ production as a sole correlate of vaccine-induced protection in mice [56,57,58,59,60]. Host defenses mounted in response to invasion by *Coccidioides* and other dimorphic fungal pathogens, including *Blastomyces* and *Histoplama*, are largely Th1-driven and disease exacerbation is a consequence of an imbalance between Th2 immunity and/or IL-10 and Th1 responses [56,57,61,62,63,64]. More recently, we applied a live attenuated vaccine (∆T) to explore the nature of adaptive immunity in mice during the initial two-week period after intranasal challenge with a potentially lethal dose of *Coccidioides* spores [65]. The numbers of pulmonary Th1 and Th17 cells showed a progressive increase in vaccinated mice and corresponded with a reduction of fungal burden [30,66,67]. Profiles of cytokines detected in lung homogenates of ∆T-vaccinated mice are indicative of a mixed Th1, Th2, and Th17 immune response. The essential requirement of T helper cells for protection against *Coccidioides* infection is demonstrated in mice using genetic deletions, designed to dispense of Th1, Th2, and Th17 immunity.

**Figure 1:**
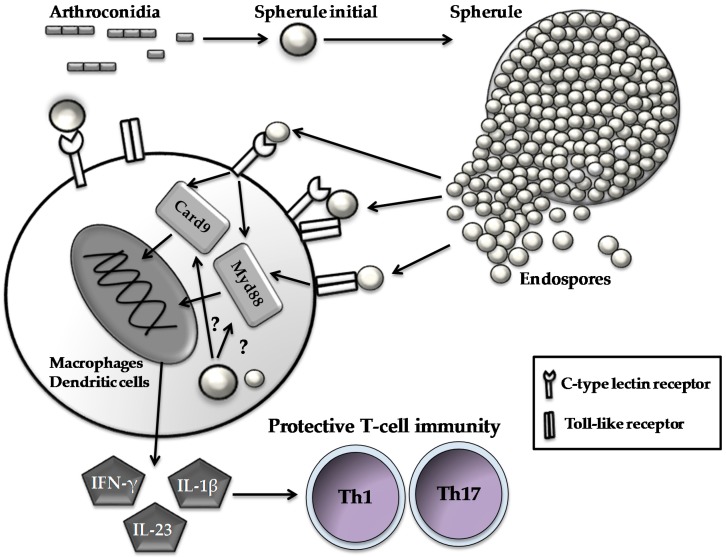
Host immune cells respond to parasitic cells of *Coccidioides*. Spherules and endospores are recognized by C-type lectin receptors and Toll-like receptors expressed on innate cell types to initiate a signal cascade via activating intracellular immune adaptors (i.e., Card9 and Myd88). Subsequently, the activated innate cells produce pro-inflammatory cytokines such as interferon-γ (FN-γ), interleukin (IL)-23 and IL-1β, which, in turn, guide the development and differentiation of Th1 and Th17 cells.

Mice lacking receptors for IFN-γ or IL-4 can still be protected against a pulmonary challenge with *Coccidioides* by vaccination with the ΔT vaccine to a degree that is equivalent to that of vaccinated wild-type counterparts. In contrast, mice lacking the IL-17 receptor can be only partially protected [30,66,67]. These data suggest that Th1 and Th17 work synergistically to eliminate *Coccidioides* infection. Studies of signal transduction pathways reveal that MyD88 and caspase recruitment domain-containing protein 9 (Card9), two intracellular immune adaptors, are essential for the activation of protective Th17 response to *Coccidioides* infection [21,23,45]. Activation of the MyD88-mediated Th17 response is dependent on the IL-1 receptor, whereas the Card9-mediated response requires Dectin-1 and Dectin-2 (Figure 1) [12,23,45]. These data support the idea that activation of Th17 and Th1 cells can enhance recruitment of phagocytes to alveoli and promote early reduction of the *Coccidioides* burden while dampening inflammatory pathology at infection sites [23,30,45,66,67,68].

T-cell responses to *Coccidioides* infection in humans share many common features with murine models of coccidioidomycosis. Humans with a deficiency in CD4^+^ T cells (i.e., HIV^+^ patients) are at elevated risk of contracting this respiratory disease [9,69]. Whole-blood samples obtained from patients with active coccidioidomycosis produce elevated levels of IL-17A after restimulation with *Coccidioides* T27K antigen compared to blood samples derived from non-immune donors [70]. IL-6 concentrations are also higher, while IL-2 and IFN-γ concentrations are significantly lower in those with disseminated coccidioidomycosis diagnosed within 12 months of disease onset relative to those with acute pneumonia. These data suggest that patients with disseminated coccidioidomycosis have an increased inflammatory response [70]. Patients with a homozygous mutation in the β1 subunit of the IL-12 receptor are predisposed to disseminated coccidioidomycosis, indicating that the IL-12/IFN-γ axis of adaptive immunity is essential for the control of *Coccidioides* infection [71]. A gain-of-function mutation in the human signal transducer and activator of transcription 1 (*STAT1*) gene results in enhanced phosphorylation, elevated DNA binding, and increased expression of IFN-γ and IFN-γ–induced genes. However, peripheral monocytic cells isolated from these patients have impaired responses to IFN-γ restimulation, likely due to unbalanced regulation of IFN-γ–mediated immunity [72]. These patients are at high risk of disseminated coccidioidomycosis, as well as other chronic fungal infections. Patients with the *STAT1* mutation also have hindered the production of IL-17A, IL-17F, and IL-22 by T cells, and, therefore, these patients have impaired IL-17 immunity and are predisposed to chronic mucocutaneous candidiasis [73]. These human studies support the importance of Th1 and Th17 immunity in the control of *Coccidioides* infection.

## 6. Development of a Multivalent Vaccine against *Coccidioides* Infection

Patients who recover from symptomatic coccidioidomycosis can acquire long-term immunity. It suggests that the development of a prophylactic vaccine against *Coccidioides* infection is feasible [57,74]. Despite the apparent ability of a live, attenuated vaccine to elicit and maintain long-term T-cell memory to *Coccidioides* infection, the ΔT vaccine may not be safe for individuals with underlying conditions of compromised cell-mediated immune systems [75]. The generation of recombinant subunit proteins may be an alternative strategy for the design of a clinically acceptable *Coccidioides* vaccine that is safe and effective [76,77]. The majority of protective antigens that have been characterized to date are products of spherules [57]. The most promising of these include two wall-associated and one intracellular antigen [78,79,80,81,82,83]. They are Antigen 2, also known as proline-rich antigen (Ag2/Pra), *Coccidioides*-specific antigen (Csa), and intracellular peroxisomal matrix protein 1 (Pmp1). These three antigens are expressed in all developing stages of spherules. Ag2/Pra and Csa are also differentially upregulated during the parasitic life cycle. Ag2/Pra is most abundant on mature spherules [84], while Csa is highly expressed on mature spherules and endospores. In order to expand coccidioidal antigen repertoires, we have applied an immunoproteomics/bioinformatics approach to identify a set of *Coccidioides* cell-wall–associated proteins, which are predicted to contain human MHC II–binding epitopes [85]. Among these antigens, aspartyl protease 1 (Pep1), phospholipase B (Plb), and α-1,2-mannosidase (Amn1) are all highly expressed during the initial stages of spherule development [86].

Compelling evidence suggests that multivalent vaccines are more potent against pulmonary *Coccidioides* infection than a vaccine containing a single peptide antigen [82,85,87]. Vaccination with a combination of three selected antigens (rPep1, rPlb, and rAmn1) provides enhanced protection against a potentially lethal, intranasal challenge, compared to immunization with one of the three antigens alone [85]. Studies have also revealed that C57BL/6 mice, vaccinated with a chimeric vaccine consisting of Ag2/Pra_1–106_ tandemly linked to Csa_1–46_, had better survival rates than littermates vaccinated with a single antigen alone [82]. Furthermore, the combination of Ag2/Pra with the second member of the proline-rich protein family (Prp2) also improved protective efficacy [87]. Our recent efforts focus on the rational design of a multivalent vaccine that is comprised of several promising coccidioidal antigens, representing different states of the parasitic cycle. This type of multivalent vaccine is necessary for induction of optimal vaccine immunity to *Coccidioides* infection. Most importantly, it is predicted to stimulate a broader range of T-cell clones than single recombinant protein vaccines and, thereby, may be capable of inducing protection in an immunologically heterogeneous human population.

Recombinant peptide antigens elicit a relatively weak immune response, and, thus, require the use of adjuvants for optimal efficacy [88]. Vaccine-mediated CD4^+^ T-cell protection against *Coccidioides* infections may work best if the response is skewed, for example, to Th1 and/or Th17 immunity guided by an adjuvant [89,90]. Our reported study shows that an epitope-based vaccine entrapped in glucan particles (GPs) elicits significantly elevated antigen-specific Th1 and Th17 immunity compared to the antigen mixed with CpG plus incomplete Freund’s adjuvant (IFA) [91]. GPs are hollow, highly purified yeast cell walls, predominantly composed of β-1,3-glucan, which have been shown to stimulate both Th1 and Th17 immunity [92,93]. GPs are phagocytosed and recognized by macrophages and dendritic cells via complement receptors and Dectin-1 [92]. The GP core can accommodate various sizes of microbial antigens and adjunctive molecules to tailor immune responses [77]. The GP adjuvant and delivery platform offer multiple options for the application of molecular tools that can create an optimized human subunit vaccine. A multivalent vaccine that is composed of several *Coccidioides* antigens with an adjuvant system to stimulate the most durable Th1 and Th17 immune response is considered the most effective formulation.

## 7. Murine Models for Evaluation of Immune Responses to *Coccidioides* Infection

The murine infection model is widely used to evaluate protective immunity and vaccine efficacy due to its low cost and the large number of genetically modified mouse strains available to researchers. Similar to observations made in patients, the susceptibility and immune responses to *Coccidioides* infection in murine models vary among strains of mice. BALB/c and C57BL/6 mice are more susceptible to infection when compared to DBA/2 mice [94]. The latter strain of mice produce elevated levels of IFN-γ, a Th-1 type cytokine, while BALB/c mice produce more IL-4, a Th-2 associated cytokine, in response to *Coccidioides* infection [95]. Interestingly, DBA/2 mice express full-length Dectin-1, whereas C57BL/6 mice splice out exon 3. The removal of exon 3 truncates the stalk that connects the extracellular CTLD motif to the transmembrane and intracellular ITAM domains [46]. Upon stimulation with *Coccidioides*, TNF-α and IL-6 produced by macrophages, as well as IL-12 and IL-23 produced by DCs, are higher in DBA/2 than in C57BL/6 mice. These results suggest that susceptibility to *Coccidioides* infection in C57BL/6 mice is associated with the expression of a truncated Dectin-1 that affects the cytokine responses of macrophages and dendritic cells [46]. Furthermore, production of the anti-inflammatory cytokine, IL-10, is also associated with the susceptibility of mice to pulmonary *Coccidioides* infection [96,97]. DCs from C57BL/6 mice produce more IL-10 than DCs from DBA/2 mice, resulting in the detrimental outcome of coccidioidomycosis in C57BL/6 mice [96,97]. Taken together, the murine model permits studies of the immunological molecules involved in the chemotaxis and activation of both innate and adaptive responses to *Coccidioides* infection. We suggest that the results of these experiments will contribute significantly to the development of a human vaccine against coccidioidomycosis.

Development of a subunit vaccine for *Coccidioides* depends on the identification of antigens that can be recognized by human T-cells. The application of conventional mouse strains to screen for human epitopes and to evaluate vaccine candidates for human use may be problematic, given the differences in the major histocompatibility complex class II (MHC II)–binding properties between murine and human antigen-presenting cells. To bridge the gap, a strain of human leukocyte antigen DR4 type (HLA-DR4; DRB1*0401 allele) transgenic mice, expressing a human MHC II receptor, has been used to evaluate vaccine efficacy and protective immune response against coccidioidomycosis [91,98]. HLA-DR4 mice are able to present peptide antigens of *Coccidioides* to autologous CD4^+^ T-cells in such a way that is solely restricted by this human MHC II molecule [85]. These results suggest that the HLA-DR4 transgenic mouse strain is one of the practical animal models for preclinical evaluation of the protective efficacy of vaccine candidates, especially subunit vaccines containing human epitopes against *Coccidioides* infection [91,98]. Human MHC II molecules are highly polymorphic. Antigen-specific T-cell receptors may only bind to certain HLAs, which differ among individuals and populations. The HLA-DP4 haplotype is one of the most abundant MHC II alleles worldwide (20%–80% of the population) [99]. HLA-DP4 transgenic mice have been created and used for the identification of microbial epitopes for vaccine development [100]. It may be beneficial to further evaluate *Coccidioides* vaccines using a strain of HLA-DP4 transgenic mice to verify their broad protective efficacy.

## 8. Conclusions

*Coccidioides* species are formidable human pathogens which are able to establish a life-threatening respiratory disease in immunocompetent individuals. There is an urgent and unmet need to develop new chemotherapy and vaccination strategies against this mycosis. Evaluation of whole-cell vaccines in murine models of coccidioidomycosis has contributed to our current understanding of the required innate and adaptive immunity for protection against this respiratory disease. It appears that an effective vaccine needs to induce Th1 and Th17 immunity to *Coccidioides* to be protective against pulmonary coccidioidomycosis. The identification of adjuvants that engage the innate immune system to augment synergistic Th1 and Th17 immunity is critical for the development of a protective *Coccidioides* vaccine. The combination of the identified adjuvant with a multivalent antigen will produce the most effective *Coccidioides* vaccine.

## Abbreviations

The following abbreviations are used in this manuscript:

Amn1	α-1,2-mannosidase
Ag2/Pra	proline-rich coccidioidal antigen
Card9	caspase recruitment domain-containing protein 9
CLRs	C-type lectin receptors
CTLDs	C-type lectin-like domains
CpG	5′-cytosine-phosphate-guanine-3′ oligonucleotide
Csa	*Coccidioides* specific antigen
DCs	dendritic cells
FKS	formalin killed spherule
GP	β-glucan particles
HLA-DR4	human leukocyte antigen-DR Related
IFA	incomplete Freud’s adjuvant
iNOS	inducible nitric oxide synthase
ITAM	immune receptor tyrosine-based activation motif
MHC	major histocompatibility complex
Mincle	macrophage-inducible C-type lectin
MyD88	myeloid differentiation primary response protein 88
NODs	Nod-like receptors
NO	Nitric oxide
NOX2	nicotinamide adenine dinucleotide phosphate oxidase subunit gp91 (phox)
PAMPs	pathogen-associated molecular patterns
Pep1	aspartyl protease 1
Plb	phospholipase B
PBMCs	peripheral blood monocytic cells
PRRs	pattern recognition receptors
RNS	reactive nitrogen species
ROS	reactive oxygen species
SOWgp	spherule outer wall glycoprotein
STAT1	signal transducer and activator of transcription 1
Tc	cytotoxic T-cells
Th	T helper cells
TLRs	Toll-like receptors

## References

[1] Engelthaler D.M., Roe C.C., Hepp C.M., Teixeira M., Driebe E.M., Schupp J.M., Gade L., Waddell V., Komatsu K., Arathoon E. (2016). Local population structure and patterns of western hemisphere dispersal for *Coccidioides* spp., the fungal cause of Valley fever. MBio.

[2] Litvintseva A.P., Marsden-Haug N., Hurst S., Hill H., Gade L., Driebe E.M., Ralston C., Roe C., Barker B.M., Goldoft M. (2015). Valley fever: Finding new places for an old disease: *Coccidioides immitis* found in Washington State soil associated with recent human infection. Clin. Infect. Dis..

[3] Cole G.T., Sun S.H., Szaniszlo P.J., Harris J.L. (1985). Arthroconidium-spherule-endospore transformation in *Coccidioides immitis*. Fungal Dimorphism: With Emphasis on Fungi Pathogenic for Humans.

[4] Fisher M.C., Koenig G.L., White T.J., Taylor J.W. (2002). Molecular and phenotypic description of *Coccidioides posadasii* sp. nov., previously recognized as the non-California population of *Coccidioides immitis*. Mycologia.

[5] Levine H.B. (1961). Purification of the spherule-endospore phase of *Coccidioides immitis*. Sabouraudia.

[6] Muhammed M., Feldmesser M., Shubitz L.F., Lionakis M.S., Sil A., Wang Y., Glavis-Bloom J., Lewis R.E., Galgiani J.N., Casadevall A. (2012). Mouse models for the study of fungal pneumonia: A collection of detailed experimental protocols for the study of *Coccidioides*, *Cryptococcus*, *Fusarium*, *Histoplasma* and combined infection due to *Aspergillus-Rhizopus*. Virulence.

[7] Lewis E.R., Bowers J.R., Barker B.M. (2015). Dust devil: The life and times of the fungus that causes Valley fever. PLoS Pathog..

[8] Galgiani J.N., Ampel N.M., Blair J.E., Catanzaro A., Geertsma F., Hoover S.E., Johnson R.H., Kusne S., Lisse J., MacDonald J.D. (2016). Infectious Diseases Society of America (IDSA) clinical practice guideline for the treatment of coccidioidomycosis. Clin. Infect. Dis..

[9] Nguyen C., Barker B.M., Hoover S., Nix D.E., Ampel N.M., Frelinger J.A., Orbach M.J., Galgiani J.N. (2013). Recent advances in our understanding of the environmental, epidemiological, immunological, and clinical dimensions of coccidioidomycosis. Clin. Microbiol. Rev..

[10] Brown J., Benedict K., Park B.J., Thompson G.R. (2013). Coccidioidomycosis: Epidemiology. Clin. Epidemiol..

[11] Wheeler C., Lucas K.D., Mohle-Boetani J.C. (2015). Rates and risk factors for coccidioidomycosis among prison inmates, California, USA, 2011. Emerg. Infect. Dis..

[12] MMWR (2013). Increase in reported coccidioidomycosis—United States, 1998–2011. MMWR Morb. Mortal. Wkly. Rep..

[13] Corzo-Leon D.E., Armstrong-James D., Denning D.W. (2015). Burden of serious fungal infections in Mexico. Mycoses.

[14] Giacomazzi J., Baethgen L., Carneiro L.C., Millington M.A., Denning D.W., Colombo A.L., Pasqualotto A.C. (2016). The burden of serious human fungal infections in Brazil. Mycoses.

[15] Thompson G.R. (2011). Pulmonary coccidioidomycosis. Semin. Respir. Crit. Care Med..

[16] Sondermeyer G., Lee L., Gilliss D., Tabnak F., Vugia D. (2013). Coccidioidomycosis-associated hospitalizations, California, USA, 2000–2011. Emerg. Infect. Dis..

[17] Sondermeyer G.L., Lee L.A., Gilliss D., Vugia D.J. (2016). Coccidioidomycosis-Associated Deaths in California, 2000–2013. Public Health Rep..

[18] Drutz D.J., Huppert M. (1983). Coccidioidomycosis: Factors affecting the host-parasite interaction. J. Infect. Dis..

[19] Erwig L.P., Gow N.A. (2016). Interactions of fungal pathogens with phagocytes. Nat. Rev. Microbiol..

[20] Lee C.Y., Thompson G.R., Hastey C.J., Hodge G.C., Lunetta J.M., Pappagianis D., Heinrich V. (2015). *Coccidioides* endospores and spherules draw strong chemotactic, adhesive, and phagocytic responses by individual human neutrophils. PLoS ONE.

[21] Hung C.Y., Castro-Lopez N., Cole G.T. (2016). Card9- and MyD88-mediated gamma interferon and nitric oxide production is essential for resistance to subcutaneous *Coccidioides posadasii* infection. Infect. Immun..

[22] Galgiani J.N. (1995). Differences in oxidant release by human polymorphonuclear leukocytes produced by stimulation with different phases of *Coccidioides immitis*. J. Infect. Dis..

[23] Hung C.Y., del Pilar Jiménez-Alzate M., Gonzalez A., Wuthrich M., Klein B.S., Cole G.T. (2014). Interleukin-1 receptor but not Toll-like receptor 2 is essential for MyD88-dependent Th17 immunity to Coccidioides infection. Infect. Immun..

[24] Parkos C.A. (2016). Neutrophil-epithelial interactions: A double-edged sword. Am. J. Pathol..

[25] Mantovani A., Sica A., Sozzani S., Allavena P., Vecchi A., Locati M. (2004). The chemokine system in diverse forms of macrophage activation and polarization. Trends Immunol..

[26] Viriyakosol S., Fierer J., Brown G.D., Kirkland T.N. (2005). Innate immunity to the pathogenic fungus *Coccidioides posadasii* is dependent on Toll-like receptor 2 and Dectin-1. Infect. Immun..

[27] Viriyakosol S., Jimenez Mdel P., Gurney M.A., Ashbaugh M.E., Fierer J. (2013). Dectin-1 is required for resistance to coccidioidomycosis in mice. MBio.

[28] Beaman L., Benjamini E., Pappagianis D. (1983). Activation of macrophages by lymphokines: Enhancement of phagosome-lysosome fusion and killing of *Coccidioides immitis*. Infect. Immun..

[29] Beaman L. (1991). Effects of recombinant gamma interferon and tumor necrosis factor on in vitro interactions of human mononuclear phagocytes with *Coccidioides immitis*. Infect. Immun..

[30] Hung C.Y., Gonzalez A., Wuthrich M., Klein B.S., Cole G.T. (2011). Vaccine immunity to coccidioidomycosis occurs by early activation of three signal pathways of T helper cell response (Th1, Th2, and Th17). Infect. Immun..

[31] Gonzalez A., Hung C.Y., Cole G.T. (2011). Nitric oxide synthase activity has limited influence on the control of *Coccidioides infection* in mice. Microb. Pathog..

[32] Gonzalez A., Hung C.Y., Cole G.T. (2011). Absence of phagocyte NADPH oxidase 2 leads to severe inflammatory response in lungs of mice infected with *Coccidioides*. Microb. Pathog..

[33] Margolis D.A., Viriyakosol S., Fierer J., Kirkland T.N. (2011). The role of reactive oxygen intermediates in experimental coccidioidomycois in mice. BMC Microbiol..

[34] Cole G.T., Kirkland T.N., Franco M., Zhu S., Yuan L., Sun S.H., Hearn V.M. (1988). Immunoreactivity of a surface wall fraction produced by spherules of *Coccidioides immitis*. Infect. Immun..

[35] Hung C.Y., Yu J.J., Seshan K.R., Reichard U., Cole G.T. (2002). A parasitic phase-specific adhesin of *Coccidioides immitis* contributes to the virulence of this respiratory Fungal pathogen. Infect. Immun..

[36] Hung C.Y., Seshan K.R., Yu J.J., Schaller R., Xue J., Basrur V., Gardner M.J., Cole G.T. (2005). A metalloproteinase of *Coccidioides posadasii* contributes to evasion of host detection. Infect. Immun..

[37] Gonzalez A., Hung C.Y., Cole G.T. (2011). *Coccidioides* releases a soluble factor that suppresses nitric oxide production by murine primary macrophages. Microb. Pathog..

[38] Roy R.M., Klein B.S. (2012). Dendritic cells in antifungal immunity and vaccine design. Cell Host Microbe.

[39] Richards J.O., Ampel N.M., Galgiani J.N., Lake D.F. (2001). Dendritic cells pulsed with *Coccidioides immitis* lysate induce antigen-specific naive T cell activation. J. Infect. Dis..

[40] Dionne S.O., Podany A.B., Ruiz Y.W., Ampel N.M., Galgiani J.N., Lake D.F. (2006). Spherules derived from *Coccidioides posadasii* promote human dendritic cell maturation and activation. Infect. Immun..

[41] Richards J.O., Ampel N.M., Lake D.F. (2002). Reversal of coccidioidal anergy in vitro by dendritic cells from patients with disseminated coccidioidomycosis. J. Immunol..

[42] Awasthi S., Awasthi V., Magee D.M., Coalson J.J. (2005). Efficacy of antigen 2/proline-rich antigen cDNA-transfected dendritic cells in immunization of mice against *Coccidioides posadasii*. J. Immunol..

[43] Vilekar P., Awasthi V., Lagisetty P., King C., Shankar N., Awasthi S. (2010). In vivo trafficking and immunostimulatory potential of an intranasally-administered primary dendritic cell-based vaccine. BMC Immunol..

[44] Plato A., Hardison S.E., Brown G.D. (2015). Pattern recognition receptors in antifungal immunity. Semin. Immunopathol..

[45] Wang H., LeBert V., Hung C.Y., Galles K., Saijo S., Lin X., Cole G.T., Klein B.S., Wuthrich M. (2014). C-type lectin receptors differentially induce Th17 cells and vaccine immunity to the endemic mycosis of North America. J. Immunol..

[46] Del Pilar Jimenez A.M., Viriyakosol S., Walls L., Datta S.K., Kirkland T., Heinsbroek S.E., Brown G., Fierer J. (2008). Susceptibility to *Coccidioides* species in C57BL/6 mice is associated with expression of a truncated splice variant of Dectin-1 (Clec7a). Genes Immun..

[47] Viriyakosol S., del Pilar Jiménez M., Saijo S., Fierer J. (2014). Neither dectin-2 nor the mannose receptor is required for resistance to Coccidioides immitis in mice. Infect. Immun..

[48] Awasthi S. (2010). Susceptibility of TLR4-defective C3H/HeJ mice to *Coccidioides posadasii* infection. Med. Mycol..

[49] Dowling J.K., Mansell A. (2016). Toll-like receptors: The swiss army knife of immunity and vaccine development. Clin. Transl. Immunol..

[50] Fierer J., Waters C., Walls L. (2006). Both CD4^+^ and CD8^+^ T cells can mediate vaccine-induced protection against *Coccidioides immitis* infection in mice. J. Infect. Dis..

[51] Wuthrich M., Filutowicz H.I., Warner T., Deepe G.S., Klein B.S. (2003). Vaccine immunity to pathogenic fungi overcomes the requirement for CD4 help in exogenous antigen presentation to CD8^+^ T cells: Implications for vaccine development in immune-deficient hosts. J. Exp. Med..

[52] Nanjappa S.G., Heninger E., Wuthrich M., Gasper D.J., Klein B.S. (2012). Tc17 cells mediate vaccine immunity against lethal fungal pneumonia in immune deficient hosts lacking CD4^+^ T cells. PLoS Pathog..

[53] Nanjappa S.G., Heninger E., Wuthrich M., Sullivan T., Klein B. (2012). Protective antifungal memory CD8^+^ T cells are maintained in the absence of CD4^+^ T cell help and cognate antigen in mice. J. Clin. Investig..

[54] O’Shea J.J., Paul W.E. (2010). Mechanisms underlying lineage commitment and plasticity of helper CD4^+^ T cells. Science.

[55] Caza T., Landas S. (2015). Functional and phenotypic plasticity of CD4^+^ T cell subsets. Biomed. Res. Int..

[56] Cox R.A., Magee D.M. (2004). Coccidioidomycosis: Host response and vaccine development. Clin. Microbiol. Rev..

[57] Cole G.T., Xue J.M., Okeke C.N., Tarcha E.J., Basrur V., Schaller R.A., Herr R.A., Yu J.J., Hung C.Y. (2004). A vaccine against coccidioidomycosis is justified and attainable. Med. Mycol..

[58] Xue J., Hung C.Y., Yu J.J., Cole G.T. (2005). Immune response of vaccinated and non-vaccinated mice to *Coccidioides posadasii* infection. Vaccine.

[59] Li K., Yu J.J., Hung C.Y., Lehmann P.F., Cole G.T. (2001). Recombinant urease and urease DNA of *Coccidioides immitis* elicit an immunoprotective response against coccidioidomycosis in mice. Infect. Immun..

[60] Shubitz L.F., Dial S.M., Perrill R., Casement R., Galgiani J.N. (2008). Vaccine-induced cellular immune responses differ from innate responses in susceptible and resistant strains of mice infected with *Coccidioides posadasii*. Infect. Immun..

[61] Allendorfer R., Brunner G.D., Deepe G.S. (1999). Complex requirements for nascent and memory immunity in pulmonary histoplasmosis. J. Immunol..

[62] Wuthrich M., Warner T., Klein B.S. (2005). IL-12 is required for induction but not maintenance of protective, memory responses to *Blastomyces dermatitidis*: Implications for vaccine development in immune-deficient hosts. J. Immunol..

[63] Wuthrich M., Filutowicz H.I., Warner T., Klein B.S. (2002). Requisite elements in vaccine immunity to *Blastomyces dermatitidis:* Plasticity uncovers vaccine potential in immune-deficient hosts. J. Immunol..

[64] Cox R.A., Magee D.M. (1998). Protective immunity in coccidioidomycosis: The life cycle and biology of Coccidioides immitis. Res. Immunol..

[65] Xue J., Chen X., Selby D., Hung C.Y., Yu J.J., Cole G.T. (2009). A genetically engineered live attenuated vaccine of *Coccidioides posadasii* protects BALB/c mice against coccidioidomycosis. Infect. Immun..

[66] Wuthrich M., Gern B., Hung C.Y., Ersland K., Rocco N., Pick-Jacobs J., Galles K., Filutowicz H., Warner T., Evans M. (2011). Vaccine-induced protection against 3 systemic mycoses endemic to North America requires Th17 cells in mice. J. Clin. Investig..

[67] Wuthrich M., Hung C.Y., Gern B.H., Pick-Jacobs J.C., Galles K.J., Filutowicz H.I., Cole G.T., Klein B.S. (2011). A TCR transgenic mouse reactive with multiple systemic dimorphic fungi. J. Immunol..

[68] Hung C.Y., Hurtgen B.J., Bellecourt M., Sanderson S.D., Morgan E.L., Cole G.T. (2012). An agonist of human complement fragment C5a enhances vaccine immunity against *Coccidioides infection*. Vaccine.

[69] Galgiani J.N., Ampel N.M. (1990). *Coccidioides immitis* in patients with human immunodeficiency virus infections. Semin. Respir. Infect..

[70] Ampel N.M., Nesbit L.A., Nguyen C.T., Chavez S., Knox K.S., Johnson S.M., Pappagianis D. (2015). Cytokine profiles from antigen-stimulated whole-blood samples among patients with pulmonary or nonmeningeal disseminated coccidioidomycosis. Clin. Vaccine Immunol..

[71] Vinh D.C., Schwartz B., Hsu A.P., Miranda D.J., Valdez P.A., Fink D., Lau K.P., Long-Priel D., Kuhns D.B., Uzel G. (2011). Interleukin-12 receptor beta1 deficiency predisposing to disseminated coccidioidomycosis. Clin. Infect. Dis..

[72] Sampaio E.P., Hsu A.P., Pechacek J., Bax H.I., Dias D.L., Paulson M.L., Chandrasekaran P., Rosen L.B., Carvalho D.S., Ding L. (2013). Signal transducer and activator of transcription 1 (STAT1) gain-of-function mutations and disseminated coccidioidomycosis and histoplasmosis. J. Allergy Clin. Immunol..

[73] Liu L., Okada S., Kong X.F., Kreins A.Y., Cypowyj S., Abhyankar A., Toubiana J., Itan Y., Audry M., Nitschke P. (2011). Gain-of-function human STAT1 mutations impair IL-17 immunity and underlie chronic mucocutaneous candidiasis. J. Exp. Med..

[74] Spinello I.M., Munoz A., Johnson R.H. (2008). Pulmonary coccidioidomycosis. Semin. Respir. Crit. Care Med..

[75] Spring M., Murphy J., Nielsen R., Dowler M., Bennett J.W., Zarling S., Williams J., de la Vega P., Ware L., Komisar J. (2013). First-in-human evaluation of genetically attenuated *Plasmodium falciparum* sporozoites administered by bite of *Anopheles* mosquitoes to adult volunteers. Vaccine.

[76] Cole G.T., Hurtgen B.J., Hung C.Y. (2012). Progress toward a human vaccine against coccidioidomycosis. Curr. Fungal Infect. Rep..

[77] Cole G.T., Hung C.Y., Sanderson S.D., Hurtgen B.J., Wuthrich M., Klein B.S., Deepe G.S., Ostroff G.R., Levitz S.M. (2013). Novel strategies to enhance vaccine immunity against coccidioidomycosis. PLoS Pathog..

[78] Jiang C., Magee D.M., Quitugua T.N., Cox R.A. (1999). Genetic vaccination against *Coccidioides immitis*: Comparison of vaccine efficacy of recombinant antigen 2 and antigen 2 cDNA. Infect. Immun..

[79] Shubitz L., Peng T., Perrill R., Simons J., Orsborn K., Galgiani J.N. (2002). Protection of mice against *Coccidioides immitis* intranasal infection by vaccination with recombinant antigen 2/PRA. Infect. Immun..

[80] Abuodeh R.O., Shubitz L.F., Siegel E., Snyder S., Peng T., Orsborn K.I., Brummer E., Stevens D.A., Galgiani J.N. (1999). Resistance to *Coccidioides immitis* in mice after immunization with recombinant protein or a DNA vaccine of a proline-rich antigen. Infect. Immun..

[81] Pan S., Cole G.T. (1995). Molecular and biochemical characterization of a *Coccidioides immitis*-specific antigen. Infect. Immun..

[82] Shubitz L.F., Yu J.J., Hung C.Y., Kirkland T.N., Peng T., Perrill R., Simons J., Xue J., Herr R.A., Cole G.T. (2006). Improved protection of mice against lethal respiratory infection with *Coccidioides posadasii* using two recombinant antigens expressed as a single protein. Vaccine.

[83] Orsborn K.I., Shubitz L.F., Peng T., Kellner E.M., Orbach M.J., Haynes P.A., Galgiani J.N. (2006). Protein expression profiling of *Coccidioides posadasii* by two-dimensional differential in-gel electrophoresis and evaluation of a newly recognized peroxisomal matrix protein as a recombinant vaccine candidate. Infect. Immun..

[84] Galgiani J.N., Sun S.H., Dugger K.O., Ampel N.M., Grace G.G., Harrison J., Wieden M.A. (1992). An arthroconidial-spherule antigen of *Coccidioides immitis*: Differential expression during in vitro fungal development and evidence for humoral response in humans after infection or vaccination. Infect. Immun..

[85] Tarcha E.J., Basrur V., Hung C.Y., Gardner M.J., Cole G.T. (2006). Multivalent recombinant protein vaccine against coccidioidomycosis. Infect. Immun..

[86] Tarcha E.J., Basrur V., Hung C.Y., Gardner M.J., Cole G.T. (2006). A recombinant aspartyl protease of *Coccidioides posadasii* induces protection against pulmonary coccidioidomycosis in mice. Infect. Immun..

[87] Herr R.A., Hung C.Y., Cole G.T. (2007). Evaluation of two homologous proline-rich proteins of *Coccidioides posadasii* as candidate vaccines against coccidioidomycosis. Infect. Immun..

[88] Azmi F., Ahmad Fuaad A.A., Skwarczynski M., Toth I. (2014). Recent progress in adjuvant discovery for peptide-based subunit vaccines. Hum. Vaccine Immunother..

[89] Levitz S.M., Golenbock D.T. (2012). Beyond empiricism: Informing vaccine development through innate immunity research. Cell.

[90] Chen K., McAleer J.P., Lin Y., Paterson D.L., Zheng M., Alcorn J.F., Weaver C.T., Kolls J.K. (2011). Th17 cells mediate clade-specific, serotype-independent mucosal immunity. Immunity.

[91] Hurtgen B.J., Hung C.Y., Ostroff G.R., Levitz S.M., Cole G.T. (2012). Construction and evaluation of a novel recombinant T cell epitope-based vaccine against coccidioidomycosis. Infect. Immun..

[92] Huang H., Ostroff G.R., Lee C.K., Agarwal S., Ram S., Rice P.A., Specht C.A., Levitz S.M. (2012). Relative contributions of Dectin-1 and complement to immune responses to particulate b-glucans. J. Immunol..

[93] Huang H., Ostroff G.R., Lee C.K., Specht C.A., Levitz S.M. (2013). Characterization and optimization of the glucan particle-based vaccine platform. Clin. Vaccine Immunol..

[94] Kirkland T.N., Fierer J. (1983). Inbred mouse strains differ in resistance to lethal *Coccidioides immitis* infection. Infect. Immun..

[95] Magee D.M., Cox R.A. (1995). Roles of gamma interferon and interleukin-4 in genetically determined resistance to *Coccidioides immitis*. Infect. Immun..

[96] Fierer J. (2006). IL-10 and susceptibility to *Coccidioides immitis* infection. Trends Microbiol..

[97] Del Pilar Jiménez M., Walls L., Fierer J. (2006). High levels of interleukin-10 impair resistance to pulmonary coccidioidomycosis in mice in part through control of nitric oxide synthase 2 expression. Infect. Immun..

[98] Hurtgen B.J., Castro-Lopez N., Jimenez-Alzate M.D., Cole G.T., Hung C.Y. (2016). Preclinical identification of vaccine induced protective correlates in human leukocyte antigen expressing transgenic mice infected with *Coccidioides posadasii*. Vaccine.

[99] Gonzalez-Galarza F.F., Takeshita L.Y., Santos E.J., Kempson F., Maia M.H., da Silva A.L., Teles e Silva A.L., Ghattaoraya G.S., Alfirevic A., Jones A.R. (2015). Allele frequency net 2015 update: New features for HLA epitopes, KIR and disease and HLA adverse drug reaction associations. Nucleic Acids Res..

[100] Ru Z., Xiao W., Pajot A., Kou Z., Sun S., Maillere B., Zhao G., Ojcius D.M., Lone Y.C., Zhou Y. (2012). Development of a humanized HLA-A2.1/DP4 transgenic mouse model and the use of this model to map HLA-DP4-restricted epitopes of HBV envelope protein. PLoS ONE.

